# Influence of Deep Eutectic Solvents and Polyphenolic Extracts on the Structure and Functional Properties of Sodium Alginate Films

**DOI:** 10.3390/polym18020186

**Published:** 2026-01-09

**Authors:** Daniel Szopa, Paulina Wróbel, Julia Zwolińska, Hira Anwar, Maciej Kaniewski, Anna Witek-Krowiak

**Affiliations:** Department of Engineering and Technology of Chemical Processes, Faculty of Chemistry, Wrocław University of Science and Technology, Gdańska 7/9, 50-344 Wrocław, Poland; paulina.wrobel@pwr.edu.pl (P.W.); julia.zwolinska@pwr.edu.pl (J.Z.); hira.anwar@pwr.edu.pl (H.A.); maciej.kaniewski@pwr.edu.pl (M.K.)

**Keywords:** polysaccharide-based films, controlled release, hydrogen bonding, antimicrobial activity, sodium alginate, polyphenolic extract, NADES

## Abstract

The growing demand for biodegradable and functional packaging has driven research toward polysaccharide-based materials with improved performance. In this study, sodium alginate films were modified using natural deep eutectic solvents (NADES) and acorn polyphenolic extract to enhance their antimicrobial, mechanical, and thermal properties. The films were acquired by solvent casting and characterized through mechanical, spectroscopic, thermal, and microbiological analyses. Both NADES and the polyphenolic extract enhanced tensile strength and flexibility through additional hydrogen bonding within the alginate network, while the extract also introduced antioxidant functionality. Among all tested formulations, the A4E2 film exhibited the most balanced performance. FTIR spectra revealed hydrogen bonding between the film components, and thermogravimetric analysis showed an approximately 15 °C (F-EXT) and 20 °C (F-DES) shift in the main DTG degradation peak, indicating enhanced thermal stability. Controlled-release experiments demonstrated the gradual diffusion of phenolic compounds in aqueous, acidic, and fatty simulants, with an initial release phase within the first 6 h followed by sustained release up to 48 h, confirming the films’ suitability for various food environments. The combined modification reduced the growth of *Escherichia coli* and *Staphylococcus aureus* by 30–35%, with inhibition zone diameters reaching 27.52 ± 2.87 mm and 25.68 ± 1.52 mm, respectively, evidencing synergistic antimicrobial activity. These results highlight the potential of NADES- and extract-modified alginate films as sustainable materials for active food packaging applications.

## 1. Introduction

Growing environmental concerns and stricter regulations are accelerating the replacement of conventional plastics with sustainable packaging solutions [1]. Policies such as the EU Single-Use Plastics Directive (2019/904) [2] and standards like EN 13432 (EU) [3] or ASTM D5338 (USA) [4] promote waste reduction, recyclability, and safe biodegradation, driving innovation in eco-friendly materials [5]. Within this global transition, natural biopolymers from renewable resources, including polysaccharides (starch, cellulose, alginate, chitosan), proteins (gelatin, casein, zein), and polyesters such as polylactic acid (PLA), are increasingly recognized as viable alternatives to petrochemical plastics [6]. Their biodegradability, biocompatibility, and food safety make them highly attractive, although their practical use requires adequate barrier and mechanical properties in line with food-contact regulations (EU 1935/2004) [7]. Intensive research is therefore focused on enhancing film performance through composites, blends and functional coatings, with alginate emerging as one of the promising candidates for sustainable packaging [8].

Alginate is a polysaccharide derived from brown seaweed, consisting of mannuronic and guluronic acid units. It has attracted significant interest in food packaging due to its abundance, biodegradability, non-toxicity and ability to form transparent, flexible films easily [9]. Alginate possesses a distinctive characteristic of crosslinking with multivalent cations, such as calcium [10], enabling precise modulation of mechanical strength, flexibility and barrier characteristics. Its biocompatibility, edibility and robust adherence to food surfaces make it especially appropriate for edible coatings that prolong the shelf life of fruits, vegetables, and meats by minimizing water loss and regulating gas exchange [11]. Alginate may be combined with other biopolymers or fortified with nanoparticles to improve barrier and mechanical properties. Furthermore, it easily incorporates bioactive components, including antioxidants and antimicrobials, facilitating the creation of functional active films [12,13,14]. This corresponds with the fundamental idea of active packaging, which offers not just physical protection but also interacts with the food or its surroundings, such as by scavenging oxygen, regulating moisture, or releasing preservation agents. In this respect, plant polyphenols have garnered significant attention owing to their potent antioxidant and antibacterial effects [15]. They are abundantly found in fruit pomaces, vegetable peels, herbs and agro-industrial by-products [16]. Extracting polyphenols from these sources offers an economical source of essential compounds while also promoting circular economy initiatives. Incorporating polyphenols into biopolymer films might significantly prolong food shelf life by inhibiting oxidation and suppressing microbiological growth [17].

Effective extraction is crucial for utilizing the functional potential of polyphenols; yet traditional solvent-based techniques using ethanol, methanol, or acetone have safety and environmental issues and might damage delicate molecules. To address these challenges, more environmentally friendly extraction methods have been developed, with natural deep eutectic solvents (NADES) proving particularly successful [18]. NADES are combinations of natural hydrogen bond donors and acceptors—including choline chloride (ChCl) with organic acids, sugars, or polyols-offering stable, biodegradable and adjustable solvents [16]. In addition to eliminating volatile organics, they improve extraction efficiency, selectivity and compound stability [19], often resulting in comparable or superior polyphenol recovery compared with traditional hydroalcoholic systems [20].

The integration of NADES-derived extracts into biopolymer films offers numerous benefits. They can solubilize a broader range of polyphenols, including highly polar or unstable compounds that are often omitted in conventional extractions, thus enhancing films with a more robust combination of bioactive compounds [21,22]. Their consumable and biodegradable components mitigate concerns about solvent residues and may improve film efficacy; for instance, ChCl demonstrates antimicrobial properties [23], whereas organic acids, like citric or lactic acid (LA), can facilitate ionic crosslinking in alginate matrices [24]. NADES enhance the molecular dispersion of polyphenols within hydrophilic polymers [25], reducing aggregation. Moreover, numerous NADES function as plasticizers [26], improving flexibility, and certain types have inherent antibacterial capabilities, providing synergistic advantages. In combination, these attributes render NADES-based extracts as attractive tools for enhancing active packaging systems.

This study aims to develop a novel bioactive food packaging film based on sodium alginate enriched with polyphenols extracted from an unconventional source—acorns. Although rarely considered in food or packaging applications, acorns are rich in tannins and other phenolic compounds with strong antioxidant potential [27,28,29]. Also, acorns are a nutrient-rich raw material characterized by a high content of carbohydrates, crude proteins, and fatty acids, which contribute to their nutritional and energy value. The lipid fraction of acorns is dominated by unsaturated fatty acids such as oleic, linoleic and palmitic acids, which are widely recognized for their beneficial effects. Acorns are also rich in several phytosterols, bioactive compounds known for their functional properties and potential role in reducing intestinal cholesterol absorption. In addition to macronutrients, acorns contain significant amounts of essential minerals, including potassium, phosphorus, magnesium, and calcium, further enhancing their nutritional quality [30,31]. Previous studies have shown that acorn extracts can protect lipid-rich foods, such as chicken meat patties, from oxidative spoilage during refrigerated storage [30], which highlights their potential as natural preservatives. Despite this, acorns remain underutilized and are often regarded as forest or agricultural waste. Their use as a source of polyphenols not only provides a novel bioactive ingredient but also contributes to the valorization of biowaste within a circular economy framework.

In this work, acorn polyphenols are extracted using a natural deep eutectic solvent composed of ChCl and LA. This solvent system was selected based on previous optimization, showing its efficiency in solubilizing polyphenols from acorn matrices. Both components are food-grade; ChCl is a dietary supplement, while LA is a typical food acidulant, and the solvent offers a low environmental footprint. The literature confirms that ChCl, combined with organic acids, forms highly effective NADES for polyphenol extraction [20], which supports the choice of this method. The obtained extract, rich in polyphenols, is incorporated into sodium alginate films by solution casting, with glycerol used as a plasticizer to enhance flexibility and calcium chloride as a crosslinker to stabilize the alginate network and entrap the polyphenols.

The novelty of this research lies in integrating an underutilized phenolic source, a green extraction method, and an entirely natural film matrix. Acorns exhibit an interesting polyphenol composition that has not been thoroughly investigated in packing materials. The application of NADES ensures effective and sustainable extraction of bioactive compounds while preserving their stability, and integrating these extracts into alginate films produces biodegradable, possibly consumable materials with improved antioxidant and antibacterial characteristics. This is the first study of acorn-derived antioxidants incorporated into alginate films and one of the few to investigate NADES-extracted bioactives within a biopolymer matrix. The present research illustrates how acorn-NADES extracts can convert alginate into a value-added active packaging system that enhances food quality and promotes environmental sustainability by assessing their functional performance.

## 2. Materials and Methods

### 2.1. Materials

Sodium alginate, choline chloride, gallic acid monohydrate, and sodium carbonate were purchased from Sigma Aldrich (St. Louis, MO, USA). A commercial acorn flour was purchased from Dary Natury (Grodzisk, Poland). Lactic acid (80%), glycerol anhydrous and Folin–Ciocalteu reagent were from Chempur (Piekary Slaskie, Poland). Ethanol (99.9%) was purchased from Stanlab (Lublin, Poland). Luria Bertani (LB) Broth and LB agar were purchased from A&A Biotechnology (Gdańsk, Poland). Calcium chloride anhydrous was from EUROCHEM BGD (Tarnow, Poland). Bacterial strains (*Escherichia coli* ATCC 10536, *Pseudomonas aeruginosa* ATCC 15442 and *Staphylococcus aureus* ATCC 25923) were obtained from the American Type Culture Collection (ATCC, Manassas, VA, USA).

### 2.2. Preparation of Extract

A NADES composed of ChCl, LA and water in a molar ratio of 1:2:2 was prepared for the extraction of polyphenols from acorns. The components were heated at 60 °C and stirred until a clear, transparent liquid was obtained. The resulting NADES was subsequently diluted with water in a mass ratio of 0.75:0.25 (NADES/water). The extraction was performed using a material-to-solvent mass ratio of 1:10, at 60 °C for 60 min under continuous stirring at 300 rpm using a magnetic stirrer. After extraction, the samples were centrifuged at 6000 rpm for 10 min using a FRONTIER FC5706 centrifuge (OHAUS, Nänikon-Greifensee, Switzerland). The extract was filtered and preserved at 4 °C.

### 2.3. Selection of Optimal Films

The alginate films for matrix selection tests were prepared by dissolving sodium alginate in water at concentrations of 1, 2, or 4% *w*/*w*. Depending on the batch, acorn extract was added at concentrations of 0, 0.5, 1, 2, or 5% *w*/*w*. The films were produced without a plasticizer to assess the effect of the extract on the behavior of alginate films. After mixing, 20 g of the polymer mixture was poured onto a Petri dish with a diameter of 90 mm and left to dry at room temperature for 48 h. After this period, crosslinking was performed using a 0.2 M CaCl_2_ solution. The films were fully immersed in a 0.2 M CaCl_2_ solution for 2 min to induce ionic crosslinking. The excess solution was then removed, and the films were left to dry again at room temperature for another 48 h [31]. The obtained films were tested for mechanical properties and total content of entrapped polyphenols. These tests aimed to select the optimal film composition. All measurements were performed in triplicate (*n* = 3). The obtained results were expressed as mean ± standard deviation (SD). To allow comparative analysis across formulations, the measured parameters: total polyphenol content (TPC) released from the cut films during the swelling test, tensile strength (STR), and elongation at break (ELO) were normalized using the min-max normalization method. A composite quality index (Q) was calculated as the arithmetic mean of the normalized parameters to identify the optimal film formulation.

### 2.4. Preparation of Optimal Films

**Figure 1:**
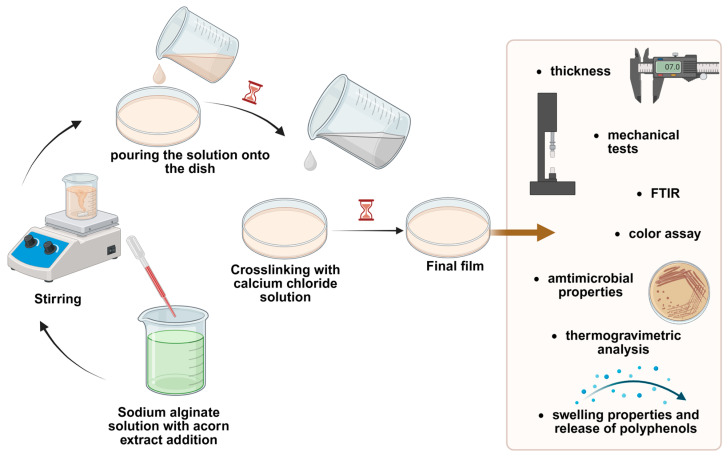
Scheme for obtaining alginate-based films containing acorn extract and evaluation of their properties. Created in BioRender. Witek-Krowiak, A. (2025) https://BioRender.com/4lgt529.

The optimal films were prepared following the same procedure as described in Section 2.3 (Selection of optimal films). Glycerol was added to the selected optimal solution before the crosslinking process to improve the properties of the matrix [32]. This modification was introduced to address difficulties in detaching the matrix from plastic Petri dishes. A total of 0.24 g of glycerol was added to 40 g of a 4% *w*/*w* sodium alginate solution. All other steps in the process remained the same as those used for obtaining alginate films (Figure 1).

### 2.5. Thickness of the Films

The thickness of the films was measured with a digital micrometer, IP65 at ten random positions on each film. Triplicate samples of each film were tested to determine film thickness.

### 2.6. Mechanical Properties

The mechanical properties of the films were determined using an INSTRON 5966 machine (INSTRON, Norwood, MA, USA). The mechanical properties of the films were evaluated under tensile loading with a crosshead pulling speed of 10 mm/min and a separation distance of 160 mm. The Young’s modulus (YM), STR, and ELO for each film were calculated along with the values for maximum compressive force (F_max_) [33,34]. Each film sample had approximate dimensions of 40 mm × 10 mm. Before each film measurement, the sample diameter was measured with a caliper. Ten repetitions of each film were taken to measure the mechanical strength.

### 2.7. Swelling Properties

The films were cut into circles, weighed and submerged in solvents at room temperature to determine the swelling kinetics at different time intervals [35]. The swollen films were removed from the solvents at predetermined times, cleaned with a paper towel to remove excess solvent and weighed. Duplicate samples of each film were tested. The amount of absorbed solvent was measured using the following Equation (1):

*Swelling* = (*Ws* − *Wi*)/*Wi* × 100, (1)

where *Ws* was the mass of the swollen film and *Wi* was the initial mass of the film.

### 2.8. Release Study

The release study of the films was determined using different simulant media: acetic media (3% acetic acid), aqueous media (water), alkaline or alcoholic, and fatty media (ethanol 10% and 50%) [36]. Briefly, the cut film (circle) was submerged in simulant media (4.5 mL). At a specific time interval, the aliquots (0.1 mL) were collected for the determination of TP release. The TPC released was periodically determined using the Folin–Ciocalteu method [32]. Release profiles were normalized to $M_{\infty }$ (taken as the maximum observed release in each medium) and fitted using the Weibull model $M_{t}/M_{\infty }=1-exp[-(t/\tau {)}^{\beta }]$ by nonlinear least-squares minimization. The shape parameter *β* was used as a diffusion/shape index, while k=1/τ was reported as an apparent release rate constant for comparison among media. For 10% ethanol, fitting was restricted to the monotonic interval (0.5–6 h) due to non-monotonic late-time values.

### 2.9. FTIR

The FTIR spectra of films were obtained using an IRAffinity-1S apparatus (Shimadzu, Kyoto, Japan) in ATR mode, with a wavenumber range of 4000–400 cm^−1^ and a resolution of 4, using 256 scans for each sample.

### 2.10. Color Assay

The color parameters (L, a, b) of the films were measured using a colorimeter (CR-400, Konica Minolta, Osaka, Japan) (D65 illuminant, 10° observer). Measurements were taken on both the matte and shiny sides at five random points per sample. The sodium alginate film served as the reference standard. All measurements were performed in triplicate.

### 2.11. Thermal Analysis

Samples were analyzed by differential scanning calorimetry coupled with thermogravimetry and mass spectrometry (DSC-TG-MS). A thermal analyzer STA 449 F3 and a mass spectrometer Aëolos QMS 403 C, (NETZSCH-Gerätebau GmbH, Selb, Germany), were used. The thermal program includes a linear heating rate equal to 10 K·min^−1^ to a temperature of 1100 °C (system reaching approximately 1040 °C) with synthetic air flow of 30 mL·min^−1^ [37,38]. Masses of approximately 5 mg of each sample were chosen for the experiment, with a 0.085 cm^3^ alumina open crucible used. Before each measurement, an empty crucible correction was performed to the appropriate temperature to compensate for thermal effects associated with properties of the crucible. All obtained results were analyzed using professional software supplied by Netzsch. During mass spectrometry, the selected ion monitoring for m/z consisted of the following signals: 15 (NH_3_), 18 (H_2_O), 30 (NO_x_), 36 (HCl), 38 (HCl, C_x_H_y_), 44 (N_2_O, CO_2_, C_x_H_2x+1_OH), 46 (NO_2_, C_x_H_2x+1_OH), and 64 (SO_2_). The meaningful *m*/*z* signals were selected from the results and presented on appropriate graphs.

### 2.12. The Antibacterial Properties

Each bacterial strain was cultured in LB broth or on LB agar. All media were sterilized by autoclaving at 121 °C for 15 min. Bacterial stocks were stored at −80 °C. To activate the cultures, a loopful of frozen stock was streaked onto LB agar plates and incubated at 37 °C for 24 h. For antimicrobial tests, bacteria were grown on Luria-Bertani broth, pH = 6.7 at 37 °C for 24 h, with constant shaking at 160 rpm [39]. After 20–24 h incubation, cultures were centrifuged at 7000 rpm for 5 min, and the supernatant was discarded. Bacterial biomass was resuspended in 5 mL sterile distilled water and the washing step was repeated twice. The optical density (OD_600_) was measured using a spectrophotometer (Biochrom WPA Biowave 3) and the suspension was then adjusted to a final concentration of 1 × 10^6^ CFU/mL for further use.

The antimicrobial activity of the films was evaluated against *E. coli*, *S. aureus*, and *P. aeruginosa* using the agar diffusion method [40]. A volume of 100 µL of bacterial inoculum, adjusted to a density of 1 × 10^6^ CFU/mL, was evenly spread onto the surface of agar plates and allowed to dry. Crude acorn extract and NADES were tested for antimicrobial activity by spotting 10 µL of each sample onto the center of agar plates previously inoculated with bacteria at a final density of 1 × 10^5^ CFU/mL. Sterile distilled water served as a negative control. For film activity, discs (approximately 25 mm) were cut, impregnated with alginate, DES, and extract, and placed at the center of the plates. All plates were incubated at 37 °C for 48 h. Inhibition zones were measured in millimeters. The experiment was performed in triplicate and the results were reported as average ± standard deviation.

## 3. Results

### 3.1. Selection of Optimal Films

**Figure 2:**
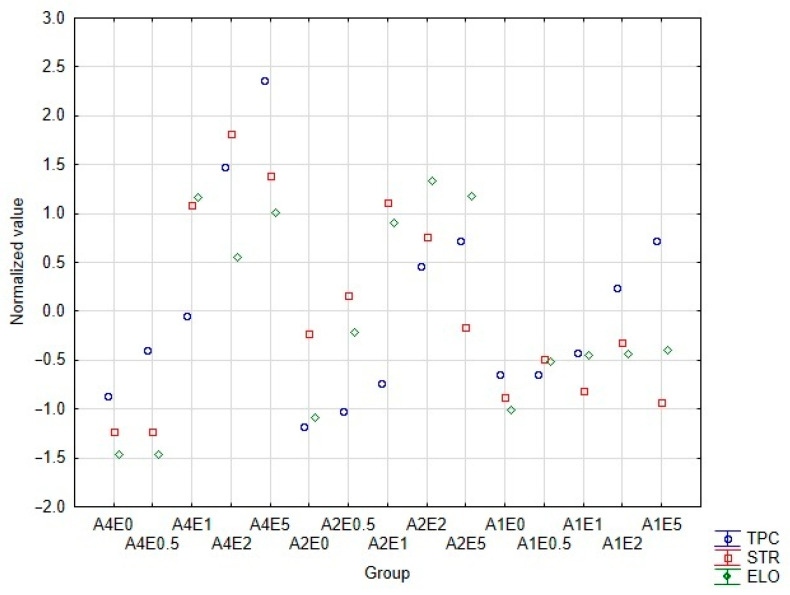
Summary of normalized parameters of films containing acorn extract.

In Figure 2, the normalized values of the qualitative factors are presented. These include TPC, STR and ELO for alginate films prepared with different concentrations of alginate (A1, A2, A4) and polyphenol extract (E0–E5). For each film formulation, three independent measurements were performed. The obtained values were averaged, and the mean values were subsequently normalized using the z-score method to allow for a comparison between parameters expressed in different units. Although each measurement was performed in triplicate, the data were used to identify general trends and select the most balanced film composition rather than to perform statistical inference. The analysis revealed nonlinear relationships between the composition and the mechanical and functional properties of the materials.

For TPC, a similar trend was observed across all alginate concentrations. As the extract concentration increased, the TPC value, which was determined by dissolving the samples in water during the swelling test, also increased. A higher alginate concentration resulted in greater encapsulation of the extract, leading to a higher overall TPC than in films with lower alginate content. The matrix demonstrated the ability to entrap active compounds. However, it has been shown that the efficiency of this process depends on the degree of crosslinking [33,34]. This observation is consistent with the literature, which reports that a higher polymer content enhances encapsulation. Less crosslinked systems may cause uneven distribution, aggregation of active substances, and their migration.

In the case of STR, an interesting tendency was observed. The addition of the polyphenolic extract did not reduce the strength of the films; instead, it increased this parameter. This effect was particularly evident for films based on 4% sodium alginate. The incorporation of 1% extract led to a significant increase in tensile strength compared to films without the extract or those containing 0.5%. It may be related to improved crosslinking. The polyphenolic extract initiated the crosslinking process after being added to the sodium alginate solution. This early interaction could have contributed to the enhancement of the structural strength of the films. The interpretation is consistent with the observation that, at lower alginate concentrations, the effect of the extract on tensile strength was less pronounced. This suggests the presence of additional hydrogen bonding between hydroxyl groups of phenols and carboxylate groups of alginate. These interactions likely increase the degree of crosslinking and improve the mechanical properties of the material [14,35]. It results from phenol-polysaccharide interactions and the antioxidant effects that stabilize the polymeric network.

The ELO parameter showed a similar trend to tensile strength. Previous studies indicate that the addition of low-molecular-weight compounds can enhance the plasticity of films. However, an excessive amount may lead to re-stiffening [36]. In the research, the addition of 5% extract did not exceed the plasticization threshold in alginate films. It is noteworthy that films with higher alginate concentrations exhibited lower mechanical properties (STR, ELO) than those with lower alginate levels. This may result from excessive packing of alginate chains, which limits efficient ionic crosslinking and induces internal stress and brittleness [37,38]. This situation does not occur at lower alginate concentrations. In that case, the crosslinking process proceeds more efficiently due to greater polymer chain mobility, which facilitates hydrogen bond formation and promotes a more uniform, less compact network structure. The preliminary results suggest that this effect arises from the absence of a plasticizer, such as glycerol, in alginate films. The aim was to determine whether the extract itself could act as a plasticizer. The results confirmed this assumption, as the mechanical behavior reversed after extract addition [35].

Based on the results, it was decided to use the A4E2 film for further research, as it provided the best balance across all quality parameters. It could be considered to use a film containing 5% of the extract. However, films with such a high extract content were non-uniform. This phenomenon is associated with exceeding the maximum amount of polyphenols that can be incorporated into the film. Moreover, the addition of the extract at this level did not significantly affect the mechanical properties of the resulting film.

### 3.2. Mechanical Properties

**Figure 3:**
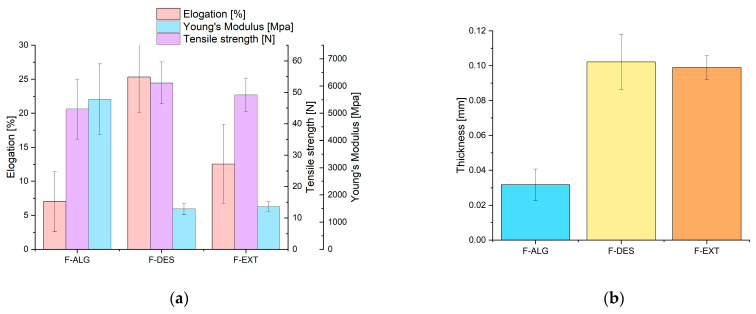

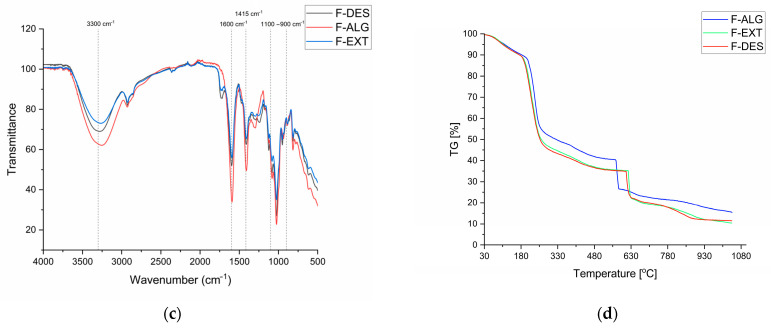
Characterization of the F-ALG, F-DES and F-EXT films: the mechanical strength of the films (**a**); the thickness of the films (**b**); the FTIR analysis of the films (**c**); the TGA of the films (**d**).

The mechanical properties, including tensile strength, ELO and YM of alginate (F-ALG), NADES-modified (F-DES) and extract-incorporated (F-EXT) films are presented in Figure 3a, and reflect the intermolecular interactions and internal structure of the polymeric matrix. As shown in the figure, the film containing pure alginate (F-ALG) exhibited the lowest ELO and the highest YM, indicating a rigid and brittle structure typical of hydrophilic alginate-based matrices. The strong intermolecular interactions within the alginate chains limit chain mobility, resulting in poor flexibility and low tensile strength [39]. The incorporation of NADES (F-DES) increased the tensile strength and ELO while reducing YM compared to the control (F-ALG). This improvement can be attributed to hydrogen bonding between NADES and alginate, which enhances polymer chain mobility and flexibility [40,41]. Such interactions can modulate the viscosity of the polymeric solution, leading to a more homogeneous and compact structure that improves mechanical stability and reduces brittleness [42]. The F-DES film exhibited the highest mechanical strength and elongation, confirming the plasticizing effect of NADES within the alginate matrix. The film containing acorn polyphenol extract (F-EXT) displayed intermediate mechanical characteristics, with tensile strength slightly lower than F-DES but higher than F-ALG. Its ELO was also higher than F-ALG’s, accompanied by a reduction in YM. These results suggest that the polyphenolic compounds of the acorn extract form hydrogen bonds with alginate chains, acting as secondary plasticizers and facilitating polymer chain relaxation [43,44]. Although these interactions may locally weaken some structural points due to the bulky polyphenolic groups acting as stress concentrators, the overall flexibility and ductility of the film were improved [45,46,47,48]. Similar findings have been reported in other studies, where the incorporation of polyphenolic compounds such as tea polyphenols, grapefruit seed extract, clove, cinnamon, star anise, and curcumin extracts into biopolymer matrices improved flexibility and mechanical toughness through hydrogen bonding and secondary interactions [9,49,50,51,52,53]. The mechanical trends observed in this study are consistent with previously reported data for alginate-based films. Only-alginate films are commonly characterized by high stiffness and low extensibility [54]. The incorporation of tannic acid into alginate-based films decreased YM and ELO with increasing tannic acid content, confirming the increase in film flexibility with polyphenol addition [55]. The use of the NADES (ChCl:LA) as a plasticizer in polysaccharide-based films has been previously reported. The higher content of NADES in chitosan-based films, the lower the tensile strength, and the higher the elongation at break, which confirms good plasticizing properties of this NADES system [56]. These results indicate that both NADES and acorn extract enhanced the ductility and toughness of alginate-based films by increasing chain mobility and reducing stiffness, making them more suitable for applications requiring flexible and resistant biopolymer materials.

### 3.3. Thickness of the Films

In Figure 3b, the thickness of the obtained films is presented. The neat alginate film (F-ALG) exhibited the lowest thickness, whereas the modified films (F-DES and F-EXT) were noticeably thicker and showed comparable mean values. This indicates that the incorporation of NADES and the polyphenolic extract influenced the film formation process, leading to the formation of thicker layers under identical casting conditions. The increased thickness of the modified films can be attributed to changes in the properties of the film-forming solutions and to altered drying behavior; the presence of NADES and extract increases the content of non-volatile components and modifies water binding, which can slow solvent evaporation and promote the formation of thicker films. Despite the higher thickness, both modified films remained mechanically stable, which is consistent with the mechanical performance shown in Figure 3a. The slightly higher variability observed for F-DES may reflect greater heterogeneity of the NADES-containing matrix during casting and drying.

### 3.4. FTIR

Figure 3c presents the FTIR spectra of the obtained films. All samples exhibit a similar spectral profile, confirming the preservation of the main polysaccharide structure. The observed differences mainly concern changes in band intensity and slight shifts in band positions. These indicate physical modifications and non-covalent interactions between the additives and sodium alginate.

**Figure 4:**
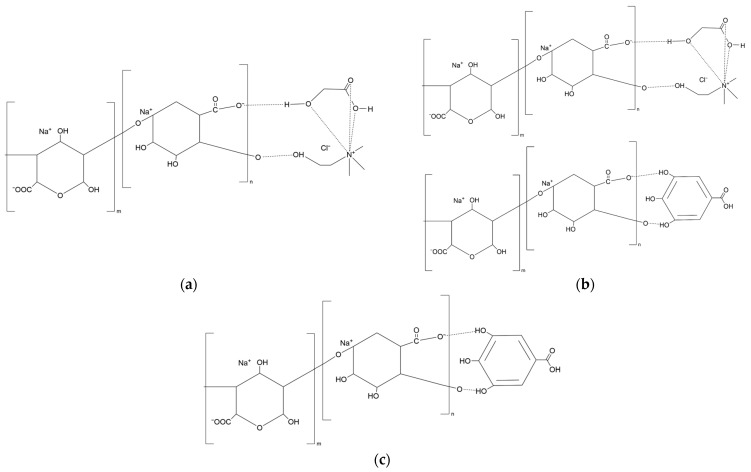
Schematic representation of possible hydrogen-bonding interactions between sodium alginate and additives: (**a**) alginate–NADES interactions, (**b**) alginate–NADES–polyphenol interactions, and (**c**) alginate–polyphenol interactions.

For the F-ALG sample, a broad band appears around 3300 cm^−1^ (Table 1). It corresponds to the stretching vibrations of hydroxyl (-OH) and carboxyl (-COOH) groups, which are typical of alginates [53,54]. Weak C-H stretching bands are visible at 2920 cm^−1^ and 2850 cm^−1^, characteristic of the polysaccharide chain. Two bands at approximately 1600 cm^−1^ and 1415 cm^−1^ originate from the carboxylate groups of the sodium alginate salt. Bands in the region of 1100–900 cm^−1^ can be assigned to C-O-C and C-O vibrations within glycosidic linkages [55,56]. The introduction of NADES (F-DES) does not lead to the appearance of new bands, indicating that the modification is physical rather than chemical. A slight decrease in the intensity of the broad O-H band and a small shift toward higher wavenumbers (around 3330 cm^−1^) are observed. This suggests the weakening of hydrogen bonds due to interactions between the -OH and -COO^−^ groups of alginate and the donor-acceptor components of the NADES [57,58]. These changes may be interpreted as a plasticizing effect, associated with partial loosening of the original hydrogen bonding network between polysaccharide chains. A schematic illustration of the possible hydrogen-bonding interactions between alginate and the additives is shown in Figure 4a–c. This observation is consistent with the mechanical property test results. In the 1600 and 1415 cm^−1^ regions, the carboxylate bands are slightly less intense than in F-ALG, which further supports the partial involvement of carboxylate groups in interactions with NADES components. The absence of distinct changes between 1400–1200 cm^−1^ and 1100–900 cm^−1^ indicates that the glycosidic structure remains undisturbed. The spectrum of the film containing the polyphenol extract (F-EXT) retains the typical alginate profile. The O-H band near 3250 cm^−1^ is slightly less intense than in the reference F-ALG sample, suggesting that hydroxyl groups from polyphenols participate in weaker and more heterogeneous hydrogen bonding [59]. In the 1600–1415 cm^−1^ range, small changes in band shape are observed, probably due to the overlap of C=O and C=C vibrations from aromatic compounds [60]. Bands between 1100 and 900 cm^−1^ remain similar to those in F-DES, suggesting a comparable, additive interaction of the extract within the glycosidic matrix.

NADES, as well as extract, do not induce chemical changes in alginate. Their presence affects only the physical reorganization of the hydrogen-bonding network, which may be related to improvements in mechanical properties. The reduction in O-H band intensity, representing the core of the alginate matrix, indicates a decrease in strong hydrogen bonding and the possible formation of weaker bonds with the added components. This effect is expected to be more pronounced in the NADES-containing film due to the higher number of donor-acceptor functional groups.

### 3.5. TGA

To confirm the weakening of hydrogen bonds and the occurrence of physical changes in the obtained F-DES and F-EXT films, thermogravimetric (TGA) analysis was performed (Figure 3d). In the case of F-ALG, a multistep decomposition pathway was observed, which is typical for alginate-based materials. The first stage was dehydration, which occurred between 40 and 195 °C, resulting in a mass loss exceeding 10% due to the removal of physically bound water [61,62]. The primary decomposition followed in the range of 195–275 °C, with a mass loss of about 36%, and was consistent with the behavior reported previously for sodium alginate [35,61,62,63,64]. Visible DSC and MS signals at *m*/*z* = 18 and 44 indicated that this process consisted of at least two partially overlapping mechanisms involving the release of H_2_O and CO_2_, together with volatile organic compounds and trace amounts of hydrochloride (*m*/*z* = 38 and 46). Between 275 and 565 °C, a gradual mass loss of approximately 13% was observed, accompanied by minor exothermic peaks on the DSC curve concurrent with the *m*/*z* = 44 signal, indicating continuous CO_2_ evolution. The TG curve showed a perceptible shift in slope above approximately 380 °C, accompanied by an additional *m*/*z* = 38 signal. This may correspond to short-chain organic fragments, as the *m*/*z* = 36 signal remained undetected for the remainder of the experiment [64,65].

The most intense stage of decomposition occurred between 565 and 585 °C, characterized by a sharp mass drop (14%) and a pronounced exothermic signal exceeding 2800 J·g^−1^, associated with the release of CO_2_, H_2_O and volatile organic compounds. It is possible that part of the Na_2_CO_3_ formed during earlier reactions also decomposed under these highly exothermic conditions, potentially in the presence of carbonaceous residues. However, the temperature observed here is somewhat lower than typically reported for Na_2_CO_3_ decomposition [66], and confirmation would require XRD analysis. This stage extended over a narrow temperature interval (20 °C) and resulted in a total mass loss exceeding 14%, consistent with the decomposition ranges (500–650 °C) reported in the literature [62,64].

Following this highly exothermic event, the residual mass underwent two additional decomposition stages. The first occurred between 585 and 670 °C, corresponding to a mass loss of approximately 3%, while the second extended up to 1030 °C, accounting for an additional 8% loss. These stages likely correspond to the gradual decomposition of residual Na_2_CO_3_ or Na_2_O to metallic sodium [66]. The final residue represented roughly 16% of the initial mass, suggesting that the predominant solid product was sodium oxide, in agreement with earlier studies [64,65].

The DTG profile of F-ALG showed a multistage degradation pattern characteristic of sodium alginate, with the main decomposition step occurring in the 200–300 °C range, followed by a high-temperature degradation event above 550 °C, in agreement with literature reports [35,61,62,64]. A small DTG peak appearing around 250 °C corresponds to the polysaccharide chain scission rather than dehydration, which occurs at significantly lower temperatures (below 150 °C). In the modified samples, the DTG peaks were shifted to higher temperatures, becoming broader and less intense, indicating a slower and more homogeneous degradation process. In F-DES, the main DTG peak shifted by about 20 °C, while for F-EXT, it shifted by approximately 15 °C, confirming enhanced thermal stability induced by both additives [35,63]. In comparison, the F-EXT and F-DES films exhibited similar multistage decomposition pathways but with distinct quantitative differences. After the dehydration step (below 180 °C), the initial decomposition occurred slightly earlier and was more pronounced, resulting in total mass losses of 42% for F-EXT and 43% for F-DES. The corresponding HCl-related MS signals (*m*/*z* = 36, 38) were more distinct, indicating a stronger presence of chloride-containing compounds. The subsequent gradual decomposition proceeded analogously to that of sodium alginate but with the final decomposition temperatures shifted upward to approximately 615 °C for F-EXT and 610 °C for F-DES. The major exothermic decomposition stage occurred like F-ALG, though at slightly elevated temperatures, with associated mass losses of 13.5% and 12.5%, respectively [35]. The final residues were 10.5% for F-EXT and 11.5% for F-DES, lower than that of pure alginate, suggesting that these samples contained additional organic compounds or that a larger fraction of Na_2_O decomposed to metallic Na, resulting in a reduced final mass [64,66]. Importantly, both T_onset_ and T_max_ values shifted to higher temperatures, and the DTG peaks broadened, particularly for F-DES, indicating enhanced thermal stability and a more gradual degradation process.

This stabilization effect originates from the formation of intermolecular hydrogen bonds between the hydroxyl and carboxyl groups of alginate and the abundant -OH and -COOH groups present in NADES components [52]. These hydrogen bridges restrict polymer chain mobility and hinder chain scission during heating. It should be noted that no new covalent bonds were detected; the stabilization arises from physical hydrogen-bonding interactions rather than chemical modifications, consistent with FTIR findings [52]. In contrast, the plant extract induced a similar but weaker effect, likely due to its lower density of hydroxyl groups and heterogeneous composition, which results in less uniform hydrogen-bond formation and a smaller increase in thermal stability [35,65].

These observations are entirely consistent with the FTIR results, in which the F-DES and F-EXT films exhibited decreased O-H band intensity and shifts in the COO^−^ stretching region, confirming the presence of hydrogen-bond interactions between alginate and the additives. The correlation between FTIR and thermal analyses demonstrates that NADES incorporation results in a more thermally stable and less brittle film structure, in line with the improved mechanical performance of the composite films.

The influence of NADES on alginate film formation is shown primarily by physical interactions rather than competitive or covalent crosslinking. FTIR analysis of F-DES films did not reveal new bands associated with esterification or permanent chemical bonding, indicating that lactic acid does not chemically crosslink alginate chains [52,57]. Instead, the observed changes in the O-H stretching region reflect a reorganization of the hydrogen-bonding network, in which NADES components partially replace strong alginate-alginate interactions with weaker, more dynamic intermolecular bonds [52,57,58]. The lactic acid may locally affect pH during casting, the final polymer network is dominated by ex situ Ca^2+^ crosslinking, ensuring that ionic interactions prevail [24]. The upward shift of T_onset_ and T_max_ in TGA, together with DTG peak broadening, confirms enhanced thermal stability arising from restricted chain mobility induced by hydrogen bonding rather than increased crosslink density [35,52,63].

### 3.6. Release and Swelling Study

**Figure 5:**
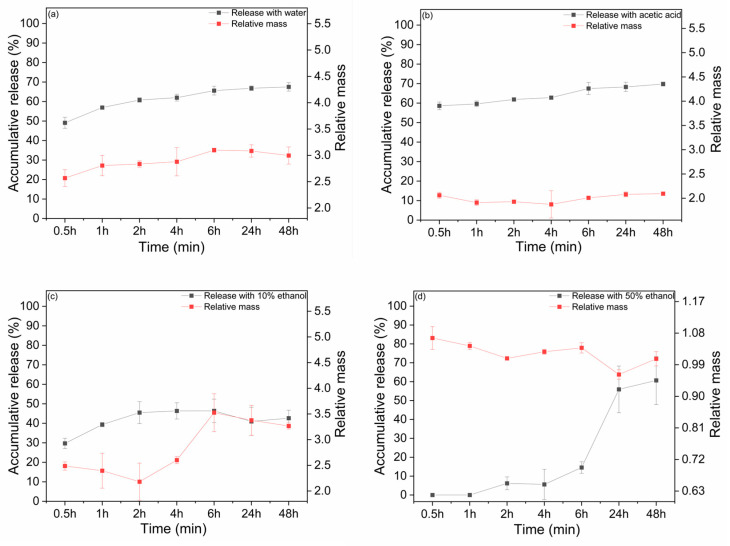
The release and swelling behaviors of the film with different simulant media: (**a**) aqueous media, (**b**) acidic media, (**c**) alcoholic media, and (**d**) fatty media.

Figure 5a–d represents the release and swelling study conducted across various simulant media, including aqueous, acidic, and fatty food, for the diffusion and release of bioactive compounds in active films. The release of the TPC from the alginate matrix is shown in Figure 5a–d from 0.5 h to 48 h, which is followed by a two-stage release: an initial burst, followed by a stable diffusion rate. The solvent molecules in different simulant media were absorbed at the film surface and then gradually penetrated the polymer matrix, causing the film to swell [67]. The highest release rate occurred primarily within the first 6 h, followed by a gradual and sustained release that extended up to 48 h across different simulant media, which showed marked differences in swelling behavior and release rates. The highest cumulative release and degree of swelling were observed in aqueous media (Figure 5a), attributed to the hydrophilic functional groups of the alginate matrix. The alginate matrix swells gradually in aqueous media (water), accelerating the rapid diffusion of TPC and confirming that water penetrates the films effectively, expanding the polymeric matrix and promoting release from the film [68]. This kind of film is favorable for a water-rich food environment, such as jellies, fruit juices, and beverages. However, a stable and constant release rate slightly lower than that of water was observed in acidic media (Figure 5b) accompanied by reduced swelling. This effect is attributed to the acidic conditions, which protonate the -COOH group of alginate, thereby decreasing the electrostatic repulsion [69]. Consequently, a compact polymeric structure formed, allowing less solvent to penetrate the polymeric matrix. As a result, the release of TPC and the degree of swelling were hindered in an acidic environment, promoting the use of acidic foods such as pickles, salad dressings and vinegar sauces.

The effect of fatty media with model ethanol (10 and 50%) solution was shown in Figure 5c,d. In Figure 5c, the results for release and swelling degree showed greater variability. The 10% ethanol solution lowers the solution’s polarity compared to water, resulting in controlled, but still noticeable, release and a degree of swelling. However, in Figure 5d with a 50% ethanol solution, the reduction is observed in both the cumulative release and the degree of swelling, where the release increases gradually over time but the swelling remains constant [70,71]. However, after 6 h, an increase in release and a decrease in mass are observed in Figure 5d. This initial slow release can be used for the intermediate/long-term protection, while after 6 h, the subsequent rapid release provides short-term protection [72]. These findings can be accompanied by the limited solubility of alginate matrix in low-polarity solvents, exhibiting a behavior similar to that of fatty food towards the environment, such as cheese, heavy and sour cream, where water penetration and diffusion rate of bioactive compounds are constrained [70]. This decrease suggested a threshold effect of ethanol concentration on the release and swelling behavior of the films. The release of polyphenols and swelling behavior observed in this study are consistent with the recent literature on alginate-based active films. Previous studies have reported a two-stage release profile with an initial burst followed by sustained diffusion, mainly noticeable in aqueous media due to the hydrophilic nature of sodium alginate matrices [73]. Alginate films incorporated with *Myrtus communis* L. leaf extract exhibited comparable release profiles in aqueous environment, while ethanol-based simulants (20–50% *v*/*v*) led to reduced swelling and slower release due to limited polymer-solvent interactions [74]. In acidic environments, alginate-based films tend to form a more compact polymeric network, which limits solvent penetration and moderately restricts matrix swelling. This behavior has been described previously and is related to pH-dependent protonation of alginate carboxyl groups, leading to reduced electrostatic repulsion between polymer chains, a phenomenon commonly reported for alginate materials [75]. Consequently, the diffusion and release of TPC in acidic media are slowed but remain stable. A quantitative comparison of the release behavior in different food simulants, the experimental release profiles were described using the Weibull model, and the resulting kinetic parameters are summarized in Table 2. Water and 3% acetic acid exhibited β values below unity, indicating burst-dominated and decelerating release behavior consistent with rapid solvent penetration and swelling of the alginate matrix. In contrast, films exposed to 50% ethanol showed β values above unity together with markedly higher characteristic times (τ), reflecting delayed and sigmoidal release associated with reduced matrix swelling and limited solvent-polymer affinity. In 10% ethanol, the early-stage release was characterized by β values close to unity, suggesting near-Fickian or mixed release behavior in the initial phase. Owing to the pronounced burst release observed at short times, the Weibull parameters should be regarded as empirical descriptors enabling comparison among simulants rather than as purely diffusion-derived constants.

Therefore, these results show that the release and swelling behavior of the acorn films indicate that the TPC release is greatly influenced by the type of selected simulant media, enhancing the usability of these films for various applications.

### 3.7. Color Assay

**Figure 6:**
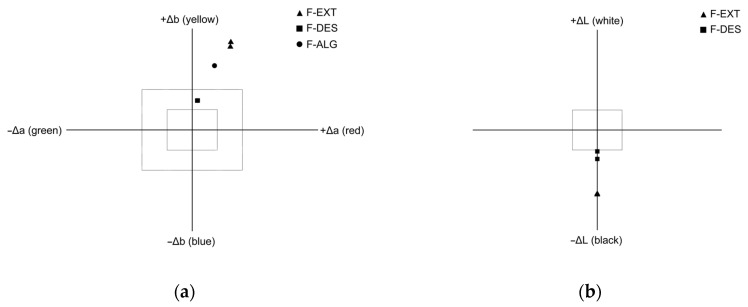
Colorimetric analysis of F-ALG, F-DES, and F-EXT films: (**a**) a*–b* chromaticity diagram showing the red–green (a*) and yellow–blue (b*) color coordinates; (**b**) L* coordinate representing lightness (white–black axis).

In Figure 6a, the color differences in alginate films are shown relative to the reference sample F-ALG. The same graph also includes the initial measurement of the reference film F-ALG, prepared from pure sodium alginate. The film exhibited slightly positive Δb values, indicating a faint yellowish hue typical of this type of material.

For the samples F-DES and F-EXT, the system was recalibrated using F-ALG as the reference. The F-DES film is located near the center of the coordinate system, indicating that the additive did not cause significant color changes compared to the pure film. A slight shift toward positive Δa and Δb values may result from minor differences in transparency between the matte and smooth sides. These differences affect light reflection but not the intrinsic color of the material.

The F-EXT film, enriched with acorn extract containing polyphenols, shows a somewhat greater shift toward positive Δa and Δb values. This corresponds to a warmer, yellow-reddish tone compared with the alginate sample. The change is moderate, confirming that the matte and smooth sides differ only slightly in color. Minor deviations may result from uneven distribution of the extract in the polymer matrix or from differences in surface roughness, both of which influence light reflection.

Figure 6b presents the brightness differences (ΔL) of the films relative to the reference sample F-ALG. Both modified films showed negative values, indicating that they were darker than the pure film. The F-DES film exhibited a slight decrease in brightness, likely due to minor changes in transparency and surface structure. The F-EXT film containing acorn extract was distinctly darker, which is consistent with the presence of colored polyphenolic compounds that absorb light. The observed trends are consistent with previous reports. Studies specify that polyphenol additives impart a slightly warmer tone to biopolymer films, while the use of NADES mainly affects gloss and transparency rather than color [76].

### 3.8. The Antibacterial Properties

The analysis of the results showed that both active components (NADES and EXT) exhibited apparent antimicrobial activity against all tested strains, including *Escherichia coli*, *Pseudomonas aeruginosa* and *Staphylococcus aureus* (Table 3). No inhibition zones were observed in the control samples or in the alginate films without additives, which confirms the absence of antimicrobial activity in these components.

The antimicrobial agents showed a weaker inhibitory effect when applied directly than when incorporated into films. The most substantial effect was observed in the F-DES group. The enhanced antimicrobial properties after incorporation into alginate matrices may be related to the prolonged presence of active compounds in direct contact with microorganisms. Slow diffusion of NADES or polyphenol molecules from the hydrogel network allows the antibacterial effect to persist throughout the incubation period. In contrast, the same compounds in solution are rapidly diluted in the agar medium. Alginate also binds water, creating a microenvironment with reduced water activity and slightly acidic pH. This enhances the action of LA present in NADES and promotes the destabilization of bacterial membranes. The hydrogel structure itself facilitates close contact between the active compounds and bacterial colonies. This increases the effectiveness of the treatment by locally raising the concentration of active molecules and extending the contact time [77,78].

The antimicrobial mechanism of NADES is associated with its acidity, low water activity, and ability to denature cellular proteins [79,80]. The activity of the acorn extract results from the presence of tannins and phenolic acids, such as gallic acid, ellagic acid and catechins, which damage cell membranes and inactivate bacterial metabolic enzymes [73,74]. The use of a biopolymer matrix enhanced the activity of both substances through physicochemical mechanisms typical of carrier systems, resulting in a significant increase in inhibition zones [75].

## 4. Future Perspectives

Future studies should focus on verifying the properties of alginate films modified with NADES and acorn extract in real food systems. Such research is necessary to confirm their stability, safety, and antimicrobial effectiveness under practical storage conditions. Particular attention should be paid to reducing the film’s affinity for water. This would help prevent excessive moisture absorption that could accelerate product spoilage or alter texture. Work is needed on matrix additives, external layers and crosslinking methods to achieve these improvements. Optimization of the formulation toward lower hydrophilicity could broaden the potential applications of these materials. Due to their biocompatibility and antimicrobial activity, such films may also be suitable for biomedical uses, including wound dressings, transdermal patches and controlled-release systems.

## 5. Conclusions

The findings confirm that the enhanced performance of alginate films stems from physical hydrogen-bond interactions between alginate, NADES, and polyphenolic compounds. These interactions improve flexibility, mechanical strength and thermal stability without altering the polymer structure. The A4E2 formulation provided the most effective balance of these properties and maintained film homogeneity, unlike samples with higher extract content that showed visible phase separation. This demonstrates that both NADES and acorn extract act synergistically as natural plasticizers and stabilizers. Their combined use not only reinforced the matrix but also introduced antioxidant and antibacterial functions, indicating a promising route toward eco-friendly, multifunctional biopolymer films for active food packaging and related sustainable applications. Future studies should evaluate the performance of the developed films under real food storage conditions and further optimize their properties for practical applications.

## Figures and Tables

**Table 1:** FTIR absorption bands of alginate-based films.

Band [cm^−1^]	Group	Observation
3600–3000 (max ~3300)	O-H stretching	Hydroxyl groups of alginate, NADES and polyphenols; hydrogen bonding
~2920 (weak, shoulder)	C-H stretching	Aliphatic C–H vibrations of the alginate backbone
~2850 (weak, shoulder)	C-H stretching	Symmetric C–H stretching
~1600	COO^−^ asymmetric stretching	Asymmetric carboxylate stretching of alginate
~1415	COO^−^ symmetric stretching	Symmetric carboxylate stretching of alginate
1400–1200	C-O/C-C vibrations	Alginate backbone vibrations
1100–900	C-O-C/C-O stretching	Glycosidic region of alginate

**Table 2:** Weibull parameters for polyphenol release from alginate films.

Medium	*M_∞_* [%]	*τ* [h]	*β*	*k = 1/τ* [h^−1^]	*t_50_* [h]	*t_90_* [h]	*R* ^2^
3% acetic acid	69.77	0.029	0.195	34.094	0.004	2.094	0.915
Water	67.51	0.220	0.364	4.545	0.080	2.180	0.976
50% ethanol	60.67	13.587	1.627	0.074	10.846	22.688	0.996
10% ethanol	46.39	0.491	0.916	2.037	0.329	1.220	0.999

**Table 3:** Data of inhibition for alginate (F-ALG), NADES (F-DES), and acorn-extract (F-EXT) films.

Organisms	Diameter of Inhibition Zone (mm)					
	Control	NADES	Extract	F-ALG	F-DES	F-EXT
*E. coli*	0.0 ± 0.0 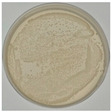	17.37 ± 1.51 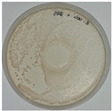	17.04 ± 0.67 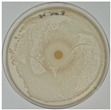	0.0 ± 0.0 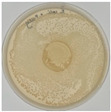	27.52± 2.87 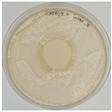	24.16 ± 1.56 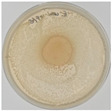
*P. aeruginosa*	0.0 ± 0.0 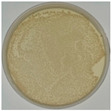	18.51 ± 0.93 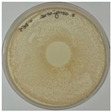	19.44 ± 0.88 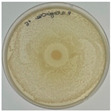	0.0 ± 0.0 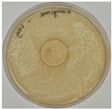	29.43 ± 1.14 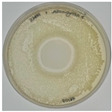	27.68 ± 1.14 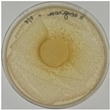
*S. aureus*	0.0 ± 0.0 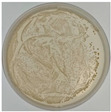	13.91 ± 0.97 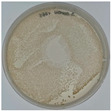	14.54 ± 1.78 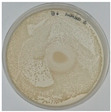	0.0 ± 0.0 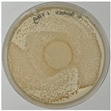	25.68 ± 1.52 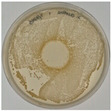	25.30 ± 0.53 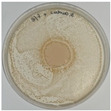

## Data Availability

The original contributions presented in this study are included in the article. Further inquiries can be directed to the corresponding authors.

## Abbreviations

The following abbreviations are used in this manuscript:

A1, A2, A4	Alginate concentrations (1%, 2%, 4%)
A4E2	Film with 4% alginate and 2% extract concentration
ChCl	Choline chloride
Δa, Δb, ΔL	Color parameters in CIELAB color space (chromaticity and lightness differences)
DSC	Differential scanning calorimetry
DTG	Derivative thermogravimetry
E0–E5	Polyphenol extract concentrations (0–5%)
ELO	Elongation at break
F-ALG	Film prepared from pure sodium alginate
F-DES	Film modified with deep eutectic solvent
F-EXT	Film incorporated with acorn polyphenol extract
FTIR	Fourier transform infrared spectroscopy
ICP-OES	Inductively coupled plasma optical emission spectrometry
LA	Lactic acid
MS	Mass spectrometry
NADES	Natural deep eutectic solvent
NMR	Nuclear magnetic resonance
STR	Tensile strength
TGA	Thermogravimetric analysis
TPC	Total polyphenol content
XRD	X-ray diffraction
YM	Young’s modulus
